# Photocurrent-induced harmonics in nanostructures

**DOI:** 10.1515/nanoph-2024-0610

**Published:** 2025-01-27

**Authors:** Ihar Babushkin, Anton Husakou, Liping Shi, Ayhan Demircan, Milutin Kovacev, Uwe Morgner

**Affiliations:** Institute of Quantum Optics, Leibniz University, Welfengarten 1, 30167 Hannover, Germany; Cluster of Excellence PhoenixD (Photonics, Optics, and Engineering-Innovation Across Disciplines), 30167 Hannover, Germany; Max Born Institute, Max Born Str. 2a, 12489 Berlin, Germany; Hangzhou Institute of Technology, Xidian University, Hangzhou 311200, China; School of Optoelectronic Engineering, Xidian University, Xi’an 710071, China

**Keywords:** Brunel harmonics, injection current harmonics, photocurrent harmonics, nanostructures, strong fields, tunnel ionization

## Abstract

Photocurrent-induced harmonics appear in gases and solids due to tunnel ionization of electrons in strong fields and subsequent acceleration. In contrast to three-step harmonic emission, no return to the parent ions is necessary. Here we show that the same mechanism produces harmonics in metallic nanostructures in strong fields. Furthermore, we demonstrate how strong local field gradient, appearing as a consequence of the field enhancement, affects photocurrent-induced harmonics. This influence can shed light at the state of electron as it appears in the continuum, in particular, to its initial velocity.

## 1 Introduction

In strong optical fields, electrons leave the surface of metallic nanostructures (NS) – the process in many respects similar to photo-induced ionization of atoms [1], [2], [3], [4]. Short, few-cycle optical pulses allow to induce sub-cycle tunneling dynamics of electrons leaving NSs [2], [3], [4], [5], [6], [7]. In the last decade this process has attracted strong and growing interest in the context of attosecond science. Most of the attention is paid to the dynamics of electrons themselves, which is of high importance in the context of generation of on-chip petahertz electronics [1], [2], [7], [8], [9], [10], scanning electron microscopy in near-field emission mode [11], and emission directly by the plasmonic fields [12]. Equally important are attempts to generate high harmonics (HHG) in the extreme ultraviolet range created by electrons returning back to the NS [13] or to the atoms in vicinity of NSs [2], [14].

In this article we consider, in contrast, a photoinduced-current mechanism. The electrons which emerge in the continuum and are subsequently accelerated by the field also emit radiation, which does not depend on their return to the parent ion [15], [16], [17], [18], [19], [20], [21]. This radiation, typically located at lower frequencies than HHG, attracted much less attention in the strong field optics, with an exception of the lowest-order (0th) harmonic. The latter is typically located in terahertz (THz) range, with photoinduced current providing a very efficient mechanism for its generation [16], [22], [23]. The higher-order photoinduced current harmonics attracted significant attention only recently [18], [19], [20], [21]. In particular, such harmonics can provide the details of redistribution of electronic wavepackets at the deeply-subcycle scale in gases [21] as well as the electron dynamics in crystal lattices [24], even if the harmonic wavelength is much larger than the atomic scale. While in the context of photoinduced current mostly the Brunel mechanism [15], [18] is considered, related to the change of the refractive index as the plasma is created, recent studies demonstrated that the creation of the free charge itself [20] (accomplished by absorbing energy from the driving pulse) is also responsible for emission of harmonics by the so-called injection current.

Here, we study the emission of both Brunel and injection-current harmonics in metallic NSs at THz and higher frequencies. We predict that the same mechanisms which acts in gases and solids will also create the photocurrent-based harmonics in the case of NSs. We show that the field gradient can significantly reduce the harmonic emission efficiency in the case if the gradient is large enough. Finally, we show that the scaling of harmonic energy with the field gradient can shed light on the dynamics of the electron wavepacket at the exit of the tunneling barrier.

The paper is structured as follows: in Section 2, we establish the semiclassical model and derive the expression for the emitted harmonics. In Section 3, we analyze the effect of the field gradient on the harmonic emission with a semiclassical model. In Section 4, the results of full quantum time-dependent Schrödinger equation (TDSE) simulation are presented, followed by a conclusion.

## 2 The system

**Figure 1:**
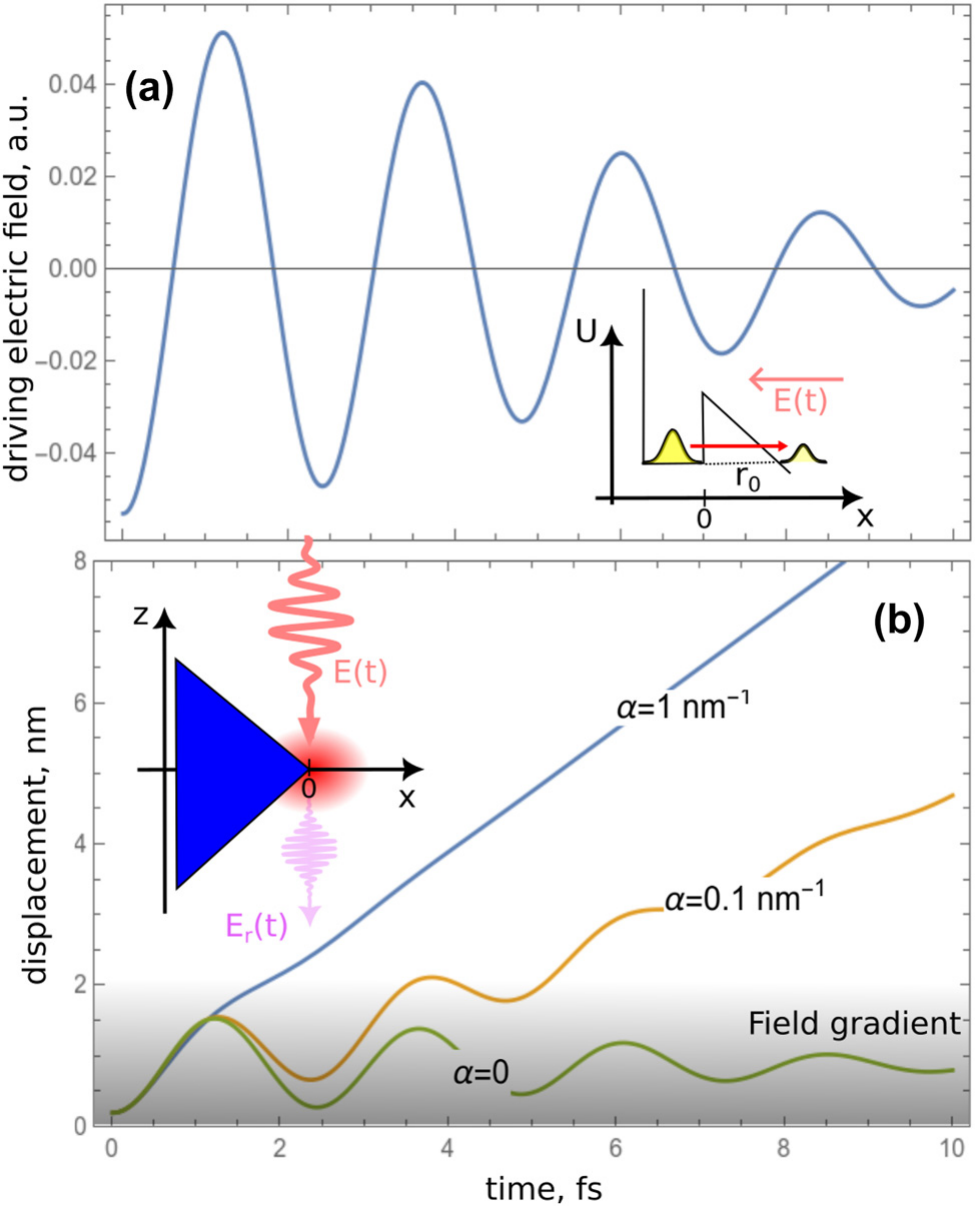
Semiclassical dynamics of electron, leaving the nanostructure. (a) An exemplary strong few-cycle pump pulse **E**(*t*) (only part of the pulse for *t* > 0 is shown), is acting on an asymmetric metallic NS (see inset in (b)). This is modeled (see inset) using the single- electron approximation with an asymmetric effective potential (thin black line) deformed by the external electric field, resulting in tunneling of the electronic wavepacket (yellow shapes, red arrow). (b) Exemplary trajectories of electrons, created at *t* = 0 near the maximum of electric field, for different values of the enhanced field gradient *α*. The inset to (b) shows the excitation geometry: the driving field **E**(*t*) leads to the field enhancement (red region) near the tip of the nanostructure (blue rectangle) and to emission of electrons, radiating the nonlinear response **E**_*r*_(*t*). It is assumed that all the fields are polarized in *x*-direction, and the movement of electrons happens also in the same direction (see inset in (a)). The gray shading in (b) illustrates schematically the field strength distribution for *α* = 1 nm^−1^.

Here we consider nanostructures, irradiated by strong few-cycle pulses (see inset in Figure 1(b)). The few-cycle duration of the pulses allows to achieve the tunnel-like photoemission process (see inset in Figure 1(a)) without immediately destroying the nanostructures [1], [2], [7]. Even if the field intensity in the vicinity of the nanostructure can approach several tens of TW/cm^2^, NSs in the few-cycle regime can still sustain milliards of pulses [25]. We focus specifically on metallic NSs, although our results are in general applicable to the dielectric NSs. The important feature of metallic NSs with sharp edges and features is a strong field enhancement near these sharp features. The enhancement mechanism is related to fast charge redistribution inside the NS and to excitation of plasmons. The field enhancement in the vicinity of the NS is localized to a small spatial region, which creates strong field gradients near the surface.

Here, we consider a small spatial region near the NS surface given by *x* = 0 (see inset in Figure 1(b)). We consider a one-dimensional geometry with all quantities depending on the spatial coordinate *x*. The pump field is polarized in *x* direction with amplitude described by

(1) $$E\left(t,x\right)=E_{0}\left(t\right)\left(1+\left(f-1\right){\text{e}}^{-\alpha x}\right)$$

for *x* > 0 (outside the NS), where the constant *α* describes the field gradient while *f* describes the field enhancement factor. Note that field polarization corresponds to the realistic situation, since the boundary conditions prevent the existence of the tangential field at the surface of an (ideal) metal. The exponential behavior of the enhancement corresponds to the spatial structure of an evanescent wave of a plasmon, which decays exponentially with the distance from the metal surface. Equation (1) is formulated in such a way that for *x* → ∞ we have *E*(*t*) → *E*_0_(*t*), whereas for *x* → 0 we have *E*(*t*) → *fE*_0_(*t*).

## 3 Semiclassical model

To gain an intuitive understanding of the relevant processes, we start from a semiclassical model, which will be followed in Section 4 by a full quantum simulation. The driving field **E**(*t*) leads to tunneling of electrons from the surface to the continuum (see inset in Figure 1(a)) and subsequent acceleration. A continuum electron (with charge −*q*) creates a dipole **P** = −*q* **r** where **r** is its position. In addition, in principle, it redistributes the electrons at the metallic NS surface, which can be described by a single or multiple effective “image” electrons. In the simplest case of flat ideal-metal surface, this would result in doubling the dipole. However, in realistic cases, the value and position of the image electron(s) depend on the geometry and the dielectric function of the NS and, on top of that, the redistribution has a finite response time. Therefore in this manuscript we prefer to disregard the image charges; for a specific NS shape and dielectric function, they can be taken into account by multiplying the dipole by a corresponding factor.

In the classical approximation we can describe the velocity **v**(*t*, *t*′) of a nonrelativistic electron at the time *t*, born at time *t*′, as

(2) $$m\frac{\text{d}v\left(t,t^{\prime }\right)}{dt}=-q\Theta \left(t-t^{\prime }\right)E\left(t,r\right),$$

where *m* is the electron mass, **E**(*t*, **r**) is the driving electric field at time *t* and position **r**. Θ(*t*) is the Heaviside step-function, describing the appearance of the electron in the continuum. The moving electron radiates the field **E**_*r*_ ∝ d**v**/d*t*. Equation (3) can be integrated, giving

(3) $$v\left(t,t^{\prime }\right)=v^{\left(0\right)}-\frac{q}{m}\overset{t}{\underset{t^{\prime }}{\int }}E\left(\tau \right)\text{d}\tau ,$$

where **v** ^(0)^ is the initial velocity of the electron at time *t*′.

The change d**P** of the total dipole **P** = −*q*∑_*i*_ **r**_*i*_, obtained by summation over all the dipoles in the continuum, is given (after approximation of the summation by integration) by

(4) $$dP\left(t\right)=-q\left(\dot{\rho }\left(t\right)r^{\left(0\right)}+\int \dot{\rho }\left(t^{\prime }\right)v\left(t,t^{\prime }\right)dt^{\prime }\right)dt,$$

where **r** ^(0)^ describes the distance from the surface at which the charges emerge in the continuum (see inset in Figure 1(a)), *ρ*(*t*) is the density of ionized electrons and $\dot{\rho }\left(t\right)$ is the ionization rate.

The first term in the right hand side of Eq. (4) describes the change of the dipole due to creation of new electrons in the continuum, whereas the second term is responsible to the modification of the dipole due to acceleration of already existing dipoles (created at earlier times *t*′). For the moment being, we have neglected the term with **v** ^(0)^ in Eq. (4).

Assuming **v**(*t*, *t*′) given by Eq. (3), and differentiating Eq. (4), we obtain the current **J** = −*q*∑_*i*_ **v**_*i*_ = d**P**/d*t* as

(5) $$\frac{\partial J}{\partial t}=-q\frac{\partial }{\partial t}\left(r^{\left(0\right)}\dot{\rho }\right)+\frac{q^{2}}{m}E\rho .$$

Finally, the radiation **E**_*r*_, observed by a remote observer, is

(6) $$E_{r}=g\frac{\partial J}{\partial t},$$

where *g* ∝ 1/*R* is a geometrical factor, depending on the distance *R* to the detector [17].

Expression Eq. (5) is analogous to that for gases and solids [17], [20]. The last contribution in Eq. (5) is known as the Brunel mechanism [15], [18] and describes the contribution to the radiation (Eq. (6)) due to acceleration of already existing dipoles, whereas the former term in the right hand side of Eq. (5) is known as injection current [20], [24] and describes the creation of the dipoles in the continuum during tunneling. Note that if no electrons are photoionized $\left(\dot{\rho }=0\right)$ , the first term in Eq. (5) is zero and the second term contributes only to the modification of the pump field phase without emission of new spectral components, since *ρ*(*t*) = const. On the other hand, $\dot{\rho }\neq 0$ leads to emission of harmonics. In addition, here we consider asymmetric structures, so both even and odd harmonics of the driving field can be created, in contrast to gases.

## 4 Impact of field gradients

**Figure 2:**
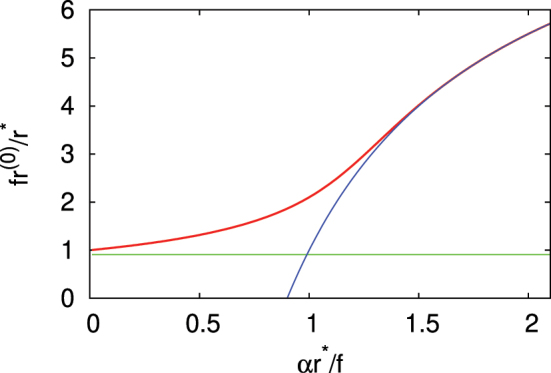
The dependence of the tunnel exit *r* ^(0)^ on the field gradient *α*. The tunnel exit (red curve) was calculated for *f* = 10. By green line, the value *r* ^(0)^ = r*/*f* is given, while by blue curve, the function *r* ^(0)^ = r* − (*f* − 1)/*α* is shown.

The tunnel exit determines the strength of the injection-current emission, which was experimentally shown to be the dominant mechanism of the low-order harmonic generation in several bulk crystals [20], [24]. It is therefore instructive and important to analyze the dependence of this quantity on the gradient of the enhanced electric field. From here on we consider linearly polarized electric field and the induced one-dimensional geometry (see insets to Figure 1), therefore for all vectorial quantities we assume the *x*-direction and use the corresponding scalar amplitudes. The tunnel exit occurs at distance *r* ^(0)^ from the NS surface in the direction of the electric field, with *r* ^(0)^ determined from the condition

(7) $$-q\overset{r^{\left(0\right)}}{\underset{0}{\int }}E\left(t,r\right)dr=U,$$

where *U* is the work function (i.e. energy necessary to remove a single electron from NS). In a homogeneous field, this condition trivially yields *r* ^(0)^ = r* ≡ −*U*/[*E*(*t*)*q*], equivalent to the known tunnel exit expression for isolated atoms and molecules. In a non-homogeneous field, such as the one given by Eq. (1), this condition is modified. In this case the tunnel exit is given by the transcendental equation

(8) $$\frac{r^{\left(0\right)}}{r^{*}}+\left(f-1\right)\frac{1-{\text{e}}^{-\alpha r^{\left(0\right)}}}{\alpha r^{*}}=1$$

which was numerically solved as shown in Figure 2. One can see that for a small inhomogeneity, the tunnel exit (red curve) is close to the value *r* ^(0)^ = r*/*f* (green line), since in this case the electron feels an almost homogeneous (enhanced) field *fE*(*t*). On the other hand, for a large inhomogeneity, the tunnel exit approaches the value *r* ^(0)^ = r* − (*f* − 1)/*α* (blue curve) which corresponds to the situation when the electron, after passing through a relatively thin layer of enhanced field *fE*(*t*), tunnels far into the space region with non-enhanced field *E*(*t*). Thus we predict the transition from enhanced-field to non-enhanced-field regime in the generation of the injection current, dependent on the field gradient.

We note that for conditions such as an intensity of 100 TW/cm^2^ and *U* of several eV, the tunnel exit is around several angstroms, which is less than the common inhomogeneity scales, which are typically a few nanometers. Tunnel exit can reach larger values for a high work function and weaker pump fields; however, it is possible that in this situation the tunneling rate is small, making the tunneling less relevant.

Let us turn our attention to the electron dynamics immediately after the photoionization. Some exemplary electron trajectories for different typical values of *α* are shown in Figure 1(b) for a 7-fs, 100-TW/cm^2^ Gaussian pulse at frequency *ω*_0_, corresponding to the central wavelength of 800 nm (see Figure 1(a) [only the region of the positive times is shown]), and gold NS with *U* = 5.1 eV. We note parenthetically that the intensity of 100 TW/cm^2^ refers to the enhanced field outside the NS; the intensity inside the NS will be much lower due to the boundary conditions, which in conjunction with the short pulse duration means that we can avoid the damage for the NS material. One can see that the gradient significantly modifies the electron trajectories. Not only the final electron velocity but also its acceleration strongly depends on *α*. In addition, the tunnel exit is visible at *t* = 0, however, it is almost independent on *α*. Since the radiation *E*_*r*_ is proportional to electron acceleration, we can expect also the modification of the photoinduced radiation by the field gradients near the NS.

One can try to solve the semiclassical model by substituting **E**(*t*, **r**) in Eq. (1) into Eq. (2) and integrating for different electron trajectories. Later on, instead of making such simulations, direct simulations of the quantum electron dynamics is performed (see the next section).

On the other hand, the semiclassical model can be further simplified to give us purely analytical insight into the dynamics. For this, we replace exp(−*αx*) in Eq. (1) by a piece-wise approximation described as

(9) $$E\left(t,x\right)=E\left(t\right)\left\{\begin{array}{cc} f,\; & \text{if}\;x<\alpha^{-1};\\ 1,\; & \text{if}\;x>\alpha^{-1}. \end{array}\right.$$

The validity of this approximation depends on the relation between, on the one hand, the maximum excursion of the electron (twice the amplitude of the spatial oscillation in the field *E*(*t*) = *fE*_0_ cos(*ω*_0_ *t*)) given by

(10) $$x_{\max}=\frac{2qfE_{0}}{m\omega_{0}^{2}},$$

and on the other hand the spatial scale of gradient *α* ^−1^. The relation between *x*_max_ and *α* ^−1^ determines how the electron “feels” the field gradient; namely, if *x*_max_ ≪ *α* ^−1^, the electron will not enter the low-field area and will not recognize any effect of the gradient. We note that the dimensionless parameter *δ* introduced in Ref. [26] has a similar role and is related to our parameters by *δ* = 2/(*αx*_max_). Let us estimate the typical values of these quantities. As can be seen from Figure 1(b), for the parameters considered in this section, *x*_max_ is around 1.5 nm. However, it can be significantly larger for stronger fields at longer wavelengths, for example, it would reach 8 nm for 2400-nm pulse with the intensity (after enhancement) of 300 TW/cm^2^. On the other hand, the scale of inhomogeneity *α* ^−1^ is typically well below the curvature radius of the sharp features of the NS [27]. In turn, these radii can in many cases reach the sub-10-nm values, which support the validity of the stepwise approximation for practical situations.

Let us estimate the time *t*″ which is required for the electron to reach *x* = *α* ^−1^ in the case *x*_max_ ≫ 1/*α*. For the evolution of the electron immediately after the ionization, which typically happens at the maximum of the electric field *E*(*t*) = −*fF*_0_, we use the second Newton’s law Eq. (2) to write

(11) $$x\left(t\right)=v^{\left(0\right)}t+\frac{fqF_{0}t^{2}}{2m}.$$

While an analytic expression for the initial electron displacement (tunnel exit) *r* ^(0)^ is readily available, the value of *v* ^(0)^ is harder to estimate and it is the topic of active discussions (see for instance [28], [29]). For large *v* ^(0)^, we get

(12) $$t^{\prime \prime }=\frac{1}{v^{\left(0\right)}\alpha },$$

while if *v* ^(0)^ can be neglected, we obtain

(13) $$t^{\prime \prime }=\sqrt{\frac{2m}{{qfF}_{0}\alpha }}.$$

The important role of *t*″ manifests itself in the scaling laws for the Brunel and injection-current harmonics. After some straightforward but tedious calculations (see Appendix A for details), we obtain

(14) $${\dot{J}}_{m}^{\text{br}}\propto t^{\prime \prime },{\dot{J}}_{m}^{\text{inj}}\propto const,$$

where ${\dot{J}}_{m}^{\text{br}}$ and ${\dot{J}}_{m}^{\text{inj}}$ are the contributions to *m*th harmonics from the Brunel and the injection current mechanisms correspondingly.

Taking into account Eq. (6) and the expressions for *t*″ above, we conclude that the contribution to the THz intensity from the Brunel mechanism is proportional to *α* ^−1^ if the initial velocity is negligible and proportional to *α* ^−2^ for the opposite case. On the other hand, the contribution from the injection current does not depend on *α* in the first approximation. These findings provide an important insight which allows to address and characterize the state of the electron immediately after ionization, as determined from *r* ^(0)^ and *v* ^(0)^. In the next section we test these scalings using direct numerical simulations of quantum equations.

## 5 Numerical simulations

To test our simple analytical theory above, we considered TDSE in the Coulomb gauge (∂_*x*_ *A* = 0):

(15) $$i\hbar \frac{\partial \psi \left(x,t\right)}{\partial t}=\frac{1}{2m}\left[{\left(p+qA\left(x,t\right)\right)}^{2}+V\left(x\right)\right]\psi \left(x,t\right),$$

where *ψ*(*x*, *t*) is the electronic wavefunction, $p=-i\hbar \frac{\partial }{\partial x}$ , *A*(*x*, *t*) is the vector potential defined in the same way as Eq. (1) with *f* = 100 and varying *α*. *V*(*x*) is a rectangular asymmetric potential

(16) $$V\left(x\right)=\left\{\begin{array}{cc} -V_{0},\; & \text{if}\;-2a<x<0;\\ 0,\; & \text{if}\;x>0;\\ \infty ,\; & \text{if}\;x<-2a, \end{array}\right.$$

where *a* = 0.106 nm, *V*_0_ = 16.94 eV were selected in such a way that (i) the potential has exactly one bound state and (ii) the ionization potential of this bound state equals to the work function of gold (5.1 eV). Even if this potential assumes that the wavefunction inside the metal is localized, the experimentally observed electron spectra [3], [4] are well-described by this rather simple approach. Therefore, it is widely used to model the ionization [1], [2], [4], [7], [26]. In the potential defined by Eq. (16), ionization can occur only in the positive direction of *x*. The electrons leaving the NS are accelerated by the field. Radiation was calculated by Eq. (6), with *g* = 1 for simplicity, and

(17) J=⟨ψ|p|ψ⟩.

The simulation was made by a split-step method, with separate evaluation of the terms ∼*p* ^2^, *pA* + *Ap*, *A* ^2^ and *V*; the action of *p* was calculated using the fast Fourier transform.

**Figure 3:**
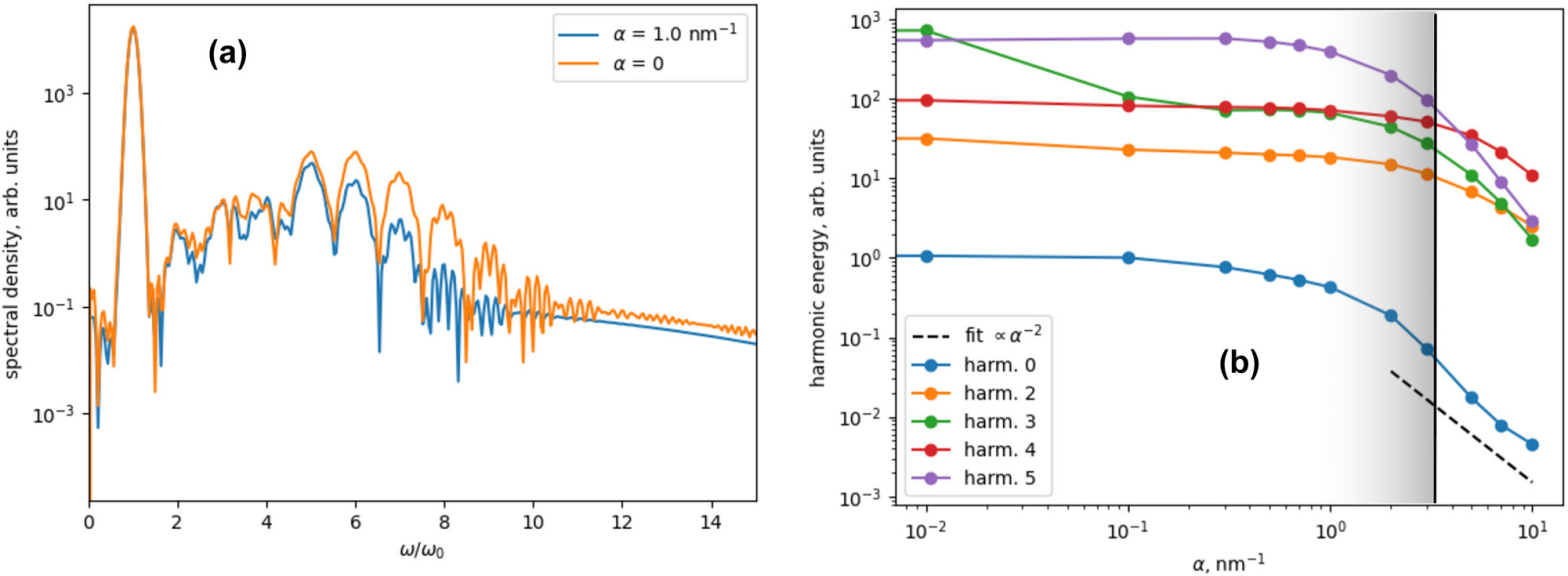
Harmonic emission and scaling with the field gradient. (a) Exemplary spectra of *E*_*r*_(*ω*) according to the TDSE simulations for a few values of the field gradient *α*. (b) The scaling of different harmonics with *α*. Black dashed line shows an exemplary fit line. Vertical black line shows *α*_bound_ = 1/*x*_max_ according to Eq. (10) (that is, for *v* ^(0)^ = 0). Shading indicates schematically the region of *α* ≈ 1/*x*_max_ for *v* ^(0)^ deviating from zero.

The results of simulations for the driving pulse with the Gaussian shape, duration of 7 fs, and amplitude *fF*_0_ = 0.053 a.u. (corresponding to the intensity of 100 TW/cm^2^) is shown in Figure 3. In Figure 3(a) the spectra of *E*_*r*_(*ω*) are shown for few exemplary values of the field gradient *α*, whereas in Figure 3(b) the dependence of the intensity of the radiated harmonics on *α* is presented. 1/*x*_max_ for *v* ^(0)^ = 0 is indicated by a vertical line. As the simple semiclassical consideration above has predicted, for small *α* ≪ 1/*x*_max_, there is almost no dependence of the radiated harmonics on *α*, with a notable exception of the third harmonic. However, as *α* approaches 1/*x*_max_, the harmonics intensity start to decrease, with the scaling approaching $\approx 1/\alpha^{2}$ for 0th harmonic and similar scaling for higher harmonics. Comparison of these results with the simple theory established in Section 3 indicates that, first, radiating pattern corresponds to the dominance of the Brunel mechanisms in this case. Second, the predicted scaling corresponds to a significant initial velocity *v* ^(0)^ of the electron wavepacket at the tunneling exit. These findings highlight the differences of the photocurrent emission in the NS case and in the case of bulk crystal, where injection current dominates the emission [20], [24]. Note, however, that these results are indirect indicators which should be used with some care and should be augmented by experimental studies (see Section 6 for more details).

We note that our semi-analytical analysis performed in the approximation of “infinitely sharp” ionization steps should provide increasingly bad estimation for the higher harmonics, especially as the period of the corresponding light approaches the ionization step duration. In this respect, it is not appropriate to consider very high harmonics (above around 7th) using the semi-analytical analysis. In particular, we see that for 5th harmonic the scaling starts to be visibly higher than $\approx 1/\alpha^{-2}$ , which can, in particular, be an indication of an increasing role of return-to-the-ion (three-step) harmonics.

## 6 Discussion and conclusions

In conclusion, here we predict that the Brunel and the injection current mechanism of harmonic emission in strong optical fields manifest themselves not only in gases but also in metallic NSs. The essential difference to the corresponding process in solids and gases originates from significant local field gradients which is typical for NS. As we showed, the field inhomogeneities start to play a notable role when the maximal excursion of the electric field *x*_max_ is of the order or larger than the inverse field gradient 1/*α*. In this case, the electron trajectories are significantly modified by the field gradient, leading to the decrease in the harmonic emission. In this sense, studying the photocurrent-induced harmonics can bring information about the local field gradients. Whereas the photo-emitted electrons are routinely detected [2], [3], [4], [5] and can provide the information about the local field gradients [5], they might be not accessible in certain cases, for instance if the nanostructure is located inside a solid or liquid.

Besides, photocurrent-induced harmonics are emitted by the electron in process of its leaving the NS, thereby imprinting the electron dynamics *in situ*. This is in contrast to imaging with electrons, which, to deliver information about the immediate vicinity of their birth in continuum, must be somehow “back-propagated” [28] from the position of their detection. To illustrate this, we have developed a semiclassical model which allows us to derive the scaling of the harmonic emission as a function of the field inhomogeneity *α*. We established that this scaling allows an additional insight into the generation mechanism and in particular into the dynamics of the electrons near the tunnel barrier exit, namely the electron velocity. In particular, in our TDSE simulations we obtained that, for large field gradients, the generation efficiency decreases approximately as *α* ^−2^, which indicates (albeit somewhat indirectly) that the Brunel mechanism dominates, and that the electronic wavepacket at the moment of the tunneling to the continuum has a non-negligible velocity. Although in our numerical simulations we scanned *α*, the quantity which matters is *x*_max_ *α*. It can be changed not only by modifying *α* (which would be difficult experimentally), but also by changing the intensity and wavelength of the driving pulse (cf. Eq. (10)).

This is consistent with the recent studies for tunneling in atoms, suggesting nonzero electron velocity at the tunnel exit [29]. Here we note that the electron wavepacket is localized neither in the momentum nor in real space, so one should speak about the initial electron velocity at the barrier exit with certain care. This equally applies to the tunneling time, that is the time, which the electron spends under the barrier, making it heavily dependent on the measurement procedure [30], [31], [32]. The same must be true also for the initial electron velocity. In our case, as mentioned above, this is the velocity which the radiated harmonics bear a fingerprint of. It must not even be identical for every particular harmonic, in the same sense as the tunneling time seen by particular recollision-based harmonic can in principle depend on the number of that harmonic [33]. In view of these unclarified questions and conceptual uncertainties it is of particular importance to design new characterization approaches for the tunneling process and pertinent electron dynamics, such as the one proposed in our paper. We note that the efficiency of the photocurrent-induced harmonics could be rather low; besides, they can be shadowed by other nonlinear mechanisms, such as strong nonlinearities in metals [34], [35], [36]. This might emerge as a challenge in the future experimental realizations. Yet, we believe that at least for the zeroth harmonic and with optimized nanostructure geometry these problems can be overcome.

We conclude that the photoinduced current-based harmonics can be generated by electron emission in NSs, not only in gases and solids. They can be furthermore used as a tool, allowing to “look inside” the initial stages of the photoemisison dynamics, and the NSs with their strong field gradients provide additional possibilities for that. In the future, when we know more about the dynamics of electrons tunneling from NSs, this approach can evolve into a tool to measure the field gradients near the nanostructures with atomic precision.

## Appendix A: Semiclassical calculation of scaling at *x*_max_ ≫ *α*

We recast the piecewise-dependent electric field as given by Eq. (9) in the following form:

(18)
$$E\left(t,x\right)=\Theta \left(t,t^{\prime },t^{\prime \prime }\left(x\right)\right)fE_{0}\left(t\right)+\Theta \left(t,t^{\prime \prime }\left(x\right),\infty \right)E_{0}\left(t\right),$$

with *t*′ being the electron birth time and *t*″(*x*) being the time moment at which the electron achieves the position *x* = *x*″ ≡ 1/*α* in the homogeneous field *E*(*t*, *x*) = *E*_0_(*t*). Here Θ(*t*, *t*_1_, *t*_2_) is a step function: Θ(*t*, *t*_1_, *t*_2_) is one of *t*_1_ < *t* < *t*_2_ and zero otherwise.

Equipped with the definitions above and taking into account the definitions of the piece-wise functions Θ(*t*, *t*_1_, *t*_2_) we can replace Eq. (2) with (assuming here both the field and velocity to be scalar):

(19)
$$\begin{array}{ll} m\dot{v} & =-qE_{0}\left(t\right)\left(f\Theta \left(t,t^{\prime },t^{\prime \prime }\right)-\Theta \left(t,t^{\prime \prime },\infty \right)\right)\\ & =m{\left(\dot{v}\right)}_{1}-m{\left(\dot{v}\right)}_{2}, \end{array}$$

where ${\left(\dot{v}\right)}_{i}$ are defined as $m{\left(\dot{v}\right)}_{i}=-qf_{i}\Theta \left(t-t_{i}^{}\right)E_{0}\left(t\right)$ with *f*_1_ = 1, *f*_2_ = *f* − 1, *t*_1_ = *t*′, *t*_2_ = *t*″.

The meaning of Eq. (19) is the following: electron acceleration can be represented a sum of two contribution of the type Eq. (2), first is from the electron born at the time *t*′ and propagating in the homogeneous field ${fE}_{0}\left(t\right)$ , and the second, with the opposite sign, from the electron born at time *t*″ and propagating in the homogeneous field (*f* − 1)*E*_0_(*t*). For *t* ≥ *t*″, we have both terms present. If *f* ≫ 1 and therefore

(20)
f−1≈f,

these terms have approximately equal amplitudes but opposite signs, and therefore cancel each other. That is, we can represent the switching off the field by spurious creation of an “electron with opposite charge sign” (one can imagine this as the creation of a positron), which contribution for the time *t* > *t*″ cancels the contribution of the already existing electron. Note that cancellation takes place if we consider acceleration $\dot{v}$ , but not for *v* itself. Yet, for the radiation, only $\dot{v}$ is important.

To apply this argument to the net growth rate of the current $\dot{J}$ in Eq. (5) (here $\dot{J}$ is the scalar version of d**J**/d*t* for our one-dimensional model), we note that, because the ionization rate $\dot{\rho }$ is the very nonlinear function of the field strength, electrons are born mostly in the vicinity of the maxima of electric field at times *t*_*n*_. Because of asymmetry of our structure, only the maxima with definite sign of *E*_0_, for instance, *E*_0_ < 0, contribute to the ionization rate. The ionization takes place in sharp steps of the typical width of order of hundred attoseconds. For this reason, as soon as we consider lowest harmonics with the oscillation period still in the femtosecond range, we can represent $\dot{\rho }$ as a sum of the delta-functions and *ρ* as sum of steps:

(21)
$$\dot{\rho }=\underset{n}{\sum }\delta \rho_{n}\left(\delta \left(t-t_{n}\right)-\delta \left(t-t_{n}-t^{\prime \prime }\right)\right),\;$$

(22)
$$\rho =\underset{n}{\sum }\delta \rho_{n}\left(\Theta \left(t-t_{n}\right)-\Theta \left(t-t_{n}-t^{\prime \prime }\right)\right),$$

where *δ*(*t*) is the Dirac *δ*-function, *δρ*_*n*_ is amplitude of the *n*th step, *t*″ is given by Eqs. (12) and (13) and corresponds to the “spurious positron” creation, which should model the inhomogeneity of the field by effectively switching it off, introduced as described above. In Eq. (21) we silently assumed Eq. (20), that is, that the field away from the NS is negligible, which makes step sizes by positive and negative terms in Eq. (21) equal, that is, negative terms completely cancel positive terms for *t* > *t*_*n*_ + *t*″.

Substituting Eqs. (21) to (5), we obtain:

(23)
$$\dot{J}=\underset{n}{\sum }\left({\dot{J}}_{n}\left(t\right)-{\dot{J}}_{n}^{\prime \prime }\left(t\right)\right),$$

(24)
$${\dot{J}}_{n}\left(t\right)=-\frac{2U\delta \rho_{n}}{\left|E\left(t_{n}\right)\right|}\dot{\delta }\left(t-t_{n}\right)+\delta \rho_{n}\frac{2q^{2}}{m}E\left(t_{n}\right)\Theta \left(t-t_{n}\right),$$

and the expression for ${\dot{J}}_{n}^{\prime \prime }\left(t\right)$ is obtained from the expression for ${\dot{J}}_{n}\left(t\right)$ by replacing *t*_*n*_ with *t*_*n*_ + *t*″.

Now we take into account that for any (sufficiently smooth) function *g*(*t*) we have

(25)
$$\begin{array}{ll} & \;g\left(t_{n}\right)\Theta \left(t-t_{n}\right)-g\left(t+t^{\prime \prime }\right)\Theta \left(t-t_{n}-t^{\prime \prime }\right)\\ & \;=g\left(t_{n}\right)\Theta \left(t,t_{n},t_{n}+t^{\prime \prime }\right)\\ & \;-\left(g_{n}^{\prime }t^{\prime \prime }+g_{n}^{\prime \prime }t^{\prime \prime 2}/2+\cdots \;\right)\times \Theta \left(t-t_{n}-t^{\prime \prime }\right), \end{array}$$

where we used the Tailor expansion of *g*(*t*) in the vicinity of *t* = *t*_*n*_ and denoted $g_{n}^{\prime }$ , $g_{n}^{\prime \prime }$ the derivatives of *g* at *t* = *t*_*n*_. Analogously,

(26)
$$\begin{array}{ll} & g\left(t_{n}\right)\dot{\delta }\left(t-t_{n}\right)-g\left(t_{n}+t^{\prime \prime }\right)\dot{\delta }\left(t-t_{n}-t^{\prime \prime }\right)\\ & \;=g\left(t_{n}\right)\dot{\delta }\left(t,t_{n},t_{n}+t^{\prime \prime }\right)\\ & \;-\left(g_{n}^{\prime }t^{\prime \prime }+g_{n}^{\prime \prime }t^{{\prime \prime }^{2}}/2+\cdots \;\right)\times \dot{\delta }\left(t-t_{n}-t^{\prime \prime }\right), \end{array}$$

where $\dot{\delta }\left(t,t_{n},t_{n}+t^{\prime \prime }\right)$ is defined as $\dot{\delta }\left(t,t_{n},t_{n}+t^{\prime \prime }\right)=\dot{\delta }\left(t-t_{n}\right)-\dot{\delta }\left(t-t_{n}-t^{\prime \prime }\right)$ . Here we assume *g*(*t*) = 2*δρ*_*n*_ *q* ^2^ *E*(*t*)/*m* for the case with Θ and *g*(*t*) = −2*Uδρ*_*n*_/|*E*(*t*)| for the case with $\dot{\delta }$ (cf. Eq. (24)). We note that since the electrons are released near the extrema of the electric field, where $g_{n}^{\prime }=0$ , only the terms with $g_{n}^{\prime \prime }$ (and higher) remain in the Tailor expansion in Eqs. (25) and (26).

Equipped with the expressions above, we can analyze the dependence of the harmonics on *α* in the region *x*_max_ ≪ *α*. Contributions from different terms to the harmonics are given by ${\dot{J}}_{m}=F\left[\dot{J}\left(t\right)\right]\left(m\omega_{0}\right)\propto \int \dot{J}{\text{e}}^{-\text{i}m\omega_{0}t}dt$ . The contribution of the term with Θ(*t*, *t*_*n*_, *t*_*n*_ + *t*″) in this integral is proportional to *t*″, the contribution of the term with Θ(*t* − *t*_*n*_ − *t*″) is proportional to $g_{n}^{\prime \prime }t^{{\prime \prime }^{2}}/\left(m\omega_{0}\right)$ , whereas the contributions from the terms with $\dot{\delta }\left(t,t_{n},t_{n}+t^{\prime \prime }\right)$ and $\dot{\delta }\left(t-t_{n}-t^{\prime \prime }\right)$ are proportional to $g_{n}^{\prime \prime }t^{{\prime \prime }^{2}}/\left(m\omega_{0}\right)$ and 1/(*mω*_0_). Keeping the leading terms in *t*″, we obtain Eq. (14).

Note that the scaling with *t*″, calculated above for every single ionization event *t*_*n*_, is readily transferred to the whole pulse. Indeed, as suggested by Eq. (23), we must sum over all ionization events *t*_*n*_. By this summation, the constructive interference takes place [17] at frequencies *ω* = *mω*_0_, recovering the standard appearance of harmonics in the emission. On the other hand, since the scaling mentioned above is valid for every particular ionization event, it is readily translated to every harmonic as a whole by the summation.

## References

[1] Vampa G. (2017). Attosecond nanophotonics. *Nat. Photonics*.

[2] Dombi P. (2020). Strong-field nano-optics. *Rev. Mod. Phys.*.

[3] Krüger M., Schenk M., Hommelhoff P. (2011). Attosecond control of electrons emitted from a nanoscale metal tip. *Nature*.

[4] Krüger M., Schenk M., Förster M., Hommelhoff P. (2012). Attosecond physics in photoemission from a metal nanotip. *J. Phys. B Atom. Mol. Opt. Phys.*.

[5] Dombi P. (2013). Ultrafast strong-field photoemission from plasmonic nanoparticles. *Nano Lett.*.

[6] Ludwig M. (2020). Sub-femtosecond electron transport in a nanoscale gap. *Nat. Phys.*.

[7] Shi L. (2021). Femtosecond field-driven on-chip unidirectional electronic currents in nonadiabatic tunnelling regime. *Laser Photonics Rev.*.

[8] Schoetz J., Wang Z., Pisanty E., Lewenstein M., Kling M. F., Ciappina M. (2019). Perspective on petahertz electronics and attosecond nanoscopy. *ACS Photonics*.

[9] Schiffrin A. (2013). Optical-field-induced current in dielectrics. *Nature*.

[10] Karnetzky C. (2018). Towards femtosecond on-chip electronics based on plasmonic hot electron nano-emitters. *Nat. Commun.*.

[11] Kirk T. L. (2017). A review of scanning electron microscopy in near field emission mode. *Adv. Imag. Electron. Phys.*.

[12] Schertz F., Schmelzeisen M., Kreiter M., Elmers H.-J., Schönhense G. (2012). Field emission of electrons generated by the near field of strongly coupled plasmons. *Phys. Rev. Lett.*.

[13] Ciappina M. F. (2014). High-order-harmonic generation driven by metal nanotip photoemission: theory and simulations. *Phys. Rev. A*.

[14] Kim S. (2008). High-harmonic generation by resonant plasmon field enhancement. *Nature*.

[15] Brunel F. (1990). Harmonic generation due to plasma effects in a gas undergoing multiphoton ionization in the high-intensity limit. *J. Opt. Soc. Am. B*.

[16] Kim K. Y., Taylor A. J., Glownia J. H., Rodriguez G. (2008). Coherent control of terahertz supercontinuum generation in ultrafast laser-gas interactions. *Nat. Photonics*.

[17] Babushkin I. (2011). Tailoring terahertz radiation by controlling tunnel photoionization events in gases. *New J. Phys.*.

[18] Babushkin I., Brée C., Dietrich C. M., Demircan A., Morgner U., Husakou A. (2017). Terahertz and higher-order Brunel harmonics: from tunnel to multiphoton ionization regime in tailored fields. *J. Mod. Opt.*.

[19] Lanin A. (2017). Mapping the electron band structure by intraband high-harmonic generation in solids. *Optica*.

[20]  Jürgens P. (2020). Origin of strong-field-induced low-order harmonic generation in amorphous quartz. *Nat. Phys.*.

[21] Babushkin I. (2022). All-optical attoclock for imaging tunnelling wavepackets. *Nat. Phys.*.

[22] Zhang X. C. (2017). Extreme terahertz science. *Nat. Photonics*.

[23] Koulouklidis A. D. (2020). Observation of extremely efficient terahertz generation from mid-infrared two-color laser filaments. *Nat. Commun.*.

[24] Jürgens P. (2024). Linking high-harmonic generation and strong-field ionization in bulk crystals. *ACS Photonics*.

[25] Shi L. (2020). Progressive self-boosting anapole-enhanced deep-ultraviolet third harmonic during few-cycle laser radiation. *ACS Photonics*.

[26] Zs Kiss G., Foldi P., Dombi P. (2022). Ultrafast plasmonic photoemission in the single-cycle and few-cycle regimes. *Sci. Rep.*.

[27] Husakou A., Im S.-J., Herrmann J. (2011). Theory of plasmon-enhanced high-order harmonic generation in the vicinity of metal nanostructures in noble gases. *Phys. Rev. A*.

[28] Ni H. (2016). Tunneling ionization time resolved by backpropagation. *Phys. Rev. Lett.*.

[29] Camus N. (2017). Experimental evidence for quantum tunneling time. *Phys. Rev. Lett.*.

[30] Landsman A. S., Keller U. (2015). Attosecond science and the tunnelling time problem. *Phys. Rep.*.

[31] Zimmermann T., Mishra S., Doran B. R., Gordon D. F., Landsman A. S. (2016). Tunneling time and weak measurement in strong field ionization. *Phys. Rev. Lett.*.

[32] Sokolovski D., Akhmatskaya E. (2018). No time at the end of the tunnel. *Commun. Phys.*.

[33] Zhao J., Lein M. (2013). Determination of ionization and tunneling times in high-order harmonic generation. *Phys. Rev. Lett.*.

[34] Shi L. (2019). Generating ultrabroadband deep-UV radiation and sub-10 nm gap by hybrid-morphology gold antennas. *Nano Lett.*.

[35] Boyd R. W., Shi Z., De Leon I. (2014). The third-order nonlinear optical susceptibility of gold. *Opt. Commun.*.

[36] Babushkin I. (2023). Metallic nanostructures as electronic billiards for nonlinear terahertz photonics. *Phys. Rev. Res.*.

